# Neuropsychiatric Symptoms as Predictors of Clinical Course in Neurodegeneration. A Longitudinal Study

**DOI:** 10.3389/fnagi.2019.00176

**Published:** 2019-07-24

**Authors:** José Manuel Santacruz Escudero, Jonathan Beltrán, Álvaro Palacios, Claudia Marcela Chimbí, Diana Matallana, Pablo Reyes, Victor Perez-Sola, Hernando Santamaría-García

**Affiliations:** ^1^Departments of Psychiatry, Physiology and Institute for Studies on the Aging, Pontificia Universidad Javeriana, Bogotá, Colombia; ^2^Intellectus Memory and Cognition Center, Hospital Universitario San Ignacio, Bogotá, Colombia; ^3^Department of Psychiatry and Forensic Medicine, Univesitat Autonòma de Bercelona, Barcelona, Spain

**Keywords:** frontotemporal dementia, Alzheimer’s disease, behavioral disturbances, depression, cohort studies, assessment of cognitive disorders/dementia

## Abstract

**Background**: To study the extent to which neuropsychiatric symptoms (NPS) influence the cognitive and functional decline in frontotemporal degeneration (FTD) and Alzheimer’s disease (AD).

**Methods**: We assessed the progression of NPS and their influence on cognitive and functional progression in a group of FTD (*n* = 36) and AD patients (*n* = 47) at two different stages of the disease (2.5 years). A standardized scale was used to assess NPS—the Columbia University Scale for Psychopathology in Alzheimer’s Disease (CUSPAD)—which tracks different symptoms including depression, psychotic symptoms, as well as sleep and conduct problems. In addition, in a subsample of patients (AD *n* = 14 and FTD *n* = 14), we analyzed another group of NPS by using the Neuropsychiatric Inventory (NPI). Cognitive declines were tracked by using the Montreal Cognitive Assessment (MoCA) and the Mini-Mental State Examination (MMSE), while functionality was tracked by using the Lawton scale and the Barthel Index.

**Results**: The presence of NPS impacts cognitive and functional decline in both groups of patients 2.5 years after disease onset. However, we observed a dissociable profile of the affectation of NPS in each group. In the AD group, results indicate that the progression of depressive symptoms and sleep problems predict cognitive and functional decline. In contrast, the progression of a mixed group of NPS, including conduct problems and delusions, predicts cognitive and functional decline in FTD.

**Conclusion**: The presence of NPS has a critical impact on the prediction of cognitive decline in FTD and AD patients after 2.5 years of disease progression. Our results demonstrate the importance of assessing different types of NPS in neurodegenerative disorders which, in turn, predict disease progression. Future studies should assess the role of NPS in predicting different neurocognitive pathways and in neurodegeneration.

## Introduction

Frontotemporal degeneration (FTD) and Alzheimer’s disease (AD) are prevalent neurodegenerative diseases which generate alterations in different cognitive and behavioral processes and have a considerable impact on the functionality of patients (Piguet et al., 2011; Livingston et al., 2017).

It is usual for patients with FTD to present alterations in the frontal, insular, and temporal brain areas, which are related to alterations in social behavior and executive functions (Piguet et al., 2011; Rascovsky et al., 2011; Sedeño et al., 2016). In turn, cognitive, language, and praxis alterations in AD have been associated with progressive atrophy in parieto-temporal areas (Dubois et al., 2014).

Both AD and FTD tend to exhibit neuropsychiatric symptoms (NPS; Ismail et al., 2016), which are considered relevant indexes for the determination of disease severity and progression (Teng et al., 2007; Peters et al., 2015). Depression and apathy have been associated with cognitive decline and mortality in AD (Teng et al., 2007; Karttunen et al., 2011; Ismail et al., 2016; Kaup et al., 2016), whereas in FTD, apathy, disinhibition and empathy impairments are the most prevalent NPS (Rascovsky et al., 2011; Brodaty et al., 2015) that impact disease progression (Brodaty et al., 2015; Ranasinghe et al., 2016). Previous studies have shown different neurocognitive progression according to the major type of symptom in FTD (Santamaría-García et al., 2016). A broad range of NPS in both AD and FTD has been described including delusions and sensory-perceptual alterations (hallucinations and illusions; Rubin et al., 1988; Van Dam et al., 2016; Gossink et al., 2017), as well as conduct problems (Santamaría-García et al., 2016; Van Dam et al., 2016), depressive and anxiety symptoms (Brodaty et al., 2015; Sellami et al., 2018), sleep problems (Mander et al., 2016; Merrilees et al., 2014) and eating changes (Ahmed et al., 2015; Ringman et al., 2015). Regarding the psychotic symptoms (including delusions and sensory-perceptive alterations such as hallucinations and illusions), previous studies have reported that those symptoms can appear in both AD and FTD. Previous studies have estimated the prevalence of psychotic symptoms in AD reached 20% and around 10% in FTD, mainly presented in patients with C9ORF72 mutations (Mendez et al., 2008). Psychotic symptoms presented in AD and FTD are associated with difficulties in episodic and working memory, poor reading of internal feelings, emotional dysregulation and inaccurate conclusions on reality associated with right frontal hypofunction and impaired activity of medial temporal areas (Mendez et al., 2008). Although the presence of psychotic symptoms has been associated with diagnosis difficulties in FTD (Velakoulis et al., 2009) and rapid cognitive decline in AD (Tchalla et al., 2018), to date, it is unknown to what extent the presence of those symptoms can impact cognitive and functionality decline in both AD and FTD.

Although previous studies have assessed the presence of different NPS in neurodegenerative disorders, to our knowledge, this is the first study assessing to what extent their presence at early stages of disease impacts the course of disease in AD and FTD.

Particularly, we study aimed to determine to what extent the presence of different types of NPS (including behavioral, affective and psychotic symptoms among others) at early stages of disease progression, predict the cognitive and functional disease progression in AD (*n* = 47) and FTD (*n* = 36) patients. Considering previous studies, we expected that depression, rather than other behavioral symptoms, has a predictive role of cognitive and functional progression in AD, but not in FTD. Conversely, we expected that the conduct problems, rather than depression, would be predictive of cognitive and functional deterioration in FTD.

## Materials and Methods

### Participants

Patients recruited to this study were divided into two groups. The first group included 36 patients who fulfilled the revised criteria for probable FTD (Rascovsky et al., 2011) and presented with prominent changes in personality and social behavior as verified by caregivers. They were recruited from the Bogotá FTD Cohort (BOGFTD), largely reported in previous studies (Baez et al., 2014a,b; Santamaría-García et al., 2016, 2017). The second group included 47 patients diagnosed with AD who were included in this study after meeting criteria outlined in McKhann et al. (2011). Patients were recruited from the Memory Clinic of the Intellectus Memory and Cognition Center, at the Hospital San Ignacio in Bogotá (Colombia). Patients underwent a standard examination battery, including neurological, neuropsychiatric, and neuropsychological assessments by geriatricians, psychiatrists, neurologists, and neuropsychologists. All patients were in the early/mild stages of the disease and did not meet the criteria for specific psychiatric disorders. Patients presenting primarily with language deficits or a history of drug abuse, or a family history of neurodegenerative or psychiatric disorders were excluded from the study.

### Initial Clinical Considerations in AD and FTD Patients

#### AD Patients

Most of the AD patients debuted with symptoms at 68.6 years and diagnosis was made on average 1.2 years after onset of symptoms (SD 0.4 years). Most of the participants debuted with cognitive symptoms consisting of episodic memory alterations (79.5%), language difficulties (43.1%) and disorientation (18.2%). A group of patients also coursed with depression (38.8%), anxiety (11, 1%), irritability (19.2%) and insomnia (12.1%).

Additionally, a group of AD patients received medications before diagnosis, including selective serotonin reuptake inhibitors (SSRIs; 12.6%), antipsychotic agents (19.4%), cholinergic agents (rivastigmine, donepezil and galantamine; 13.4%), benzodiazepines (1.5%) and GABA-A agonists (zopiclone and eszopiclone; 1.3%).

#### FTD Patients

Most of the AD patients debuted with symptoms at 60.7 years and were diagnosed 1.1 years after debut symptoms on average (SD 0.6 years). Most of the participants debuted with behavioral symptoms featured by apathy (25.9%), disinhibition (34.5%), delusions (1.9%), depression (18.5%) and anxiety (11.1%). Furthermore, 18.1% of patients coursed with attention impairments, 15.6% with language difficulties and 27. 8% with working memory impairments. A group of FTD patients received pharmacological treatments before diagnosis was made, from them 17.9% received selective serotonin reuptake inhibitors (SSRIs), 14.1% antipsychotic agents, 0.8% benzodiazepines, 0.9% valproate and 0.6% carbamazepine.

#### Assessment Milestones

Patient groups (FTD and AD) were assessed at two stages of the disease progression. Patients were assessed by the same group of specialists at both stages. All enrolled patients had the same diagnosis in the first and second stages of assessment. In the first stage, patients were assessed at the time of diagnosis. The second assessment took place 2.5 years after the first (FTD mean = 2.4 years, SD = 1.2 vs. AD mean = 2.6 years, SD = 1.1). In each stage, patients in both groups were assessed with neurocognitive measures, as well as with measures of NPS (see below). As reported in previous studies (Baez et al., 2014b; Sedeño et al., 2017), we observed differences in the age at disease onset (see Table 1).

**Table 1:** Socio-demographic description of patients diagnosed with Alzheimer’s disease (AD) and frontotemporal degeneration (FTD).

Gender	AD (*n* = 47)		FTD (*n* = 36)	
Male	20 (42.6%)		18 (50%)	
Female	27 (57.4%)		18 (50%)	
**Variables**	**Mean (SD)**	**Median**	**Mean (SD)**	**Median**
Age at time 1 (years)	72.1 (7.3)	72	64.1 (6.4)	63.4
Age at time 2 (years)	74.9 (6.2)	75	67.6 (6.9)	65
Age difference (years)	2.6 (1.1)	2	2.4 (1.2)	2
Educational level (years)	13.6 (4.9)	14	14.4 (5.2)	15
Age at disease onset (years)	68.3 (6.9)	69	61.2 (6.6)	60

*Note: SD, standard deviation; AD, Alzheimer’s disease; FTD, frontotemporal dementia*.

#### Cognitive Assessment

Global cognitive performance was assessed in two stages through a comprehensive set of measures, using the Montreal Cognitive Assessment (MoCA; Nasreddine et al., 2005; Freitas et al., 2012; Delgado et al., 2017) and the Mini-Mental State Examination (MMSE; Folstein et al., 1983). The MoCA scale has a sensitivity above 87% and a specificity of 90% with a cut-off point of 26, and a sensitivity of 87% and a specificity of 87% with a cut-off lower than 18 points. It is composed of 19 items that evaluate eight cognitive domains, including executive skills, denomination, memory, attention, language, abstraction, deferred memory and orientation (Nasreddine et al., 2005; Pedraza et al., 2016). The MMSE is a classical instrument for assessing cognitive domains, including verbal memory, working memory, language, and visuospatial functions. A score of 24 points has a sensitivity above 88.3% and a specificity close to 87% for detecting cognitive impairment in patients with neurodegeneration (Folstein et al., 1975, 1983).

#### Assessment of Instrumental Functionality

The Lawton scale is an instrument for assessing the instrumental activities of daily living (Lawton and Brody, 1969). This instrument has been used to assess the clinical progression of dementia with respect to functional commitment. It contains eight items that assess functionality, including the ability to use the phone, go shopping, prepare food, perform household care and laundry, use of public transportation, as well as medication and money management, with an internal consistency measured by a Cronbach’s alpha coefficient of 0.94 (Lawton and Brody, 1969; Vergara et al., 2012). The Lawton scale is an instrument previously reported for assessing functionality in neurodegenerative disorders (Cornelis et al., 2017).

### Assessment of Physical Functionality

In addition, we used the Barthel index (Mahoney and Barthel, 1965), which was introduced as a way to measure the impairments of patients in their neuromuscular and musculoskeletal function, placing a strong focus on the spared activity of inferior extremities. The index is an ordinal scale comprising 10 activities of daily living, including sphincter control. The original Barthel index was scored in steps of five points to give a maximum total score of 100. The Lawton scale is an instrument previously reported for assessing functionality in neurodegenerative disorders including FTD and AD (Merrilees et al., 2013; Liljegren et al., 2015).

#### Assessment of Neuropsychiatric Symptoms (NPS)

The assessment of NPS, including delusions, sensory-perceptive alterations (hallucinations and illusions), conduct problems, sleep problems, depression, anxiety, and changes in eating patterns, was conducted at two stages using the Columbia University Scale for Psychopathology in Alzheimer’s Disease (CUSPAD; Devanand et al., 1992; Suárez-González et al., 2013). CUSPAD is a quick and easy to use standardized instrument that provides information regarding neurodegenerative psychopathology. Classical reports calculated that this instrument has a high reliability (*k* = 0.74). This scale has been reported in studies assessing psychopathology in AD (Zahodne et al., 2015) and other neurodegenerative conditions (Suárez-González et al., 2013). In addition, it is an instrument that has shown high sensitivity for detecting psychosis (Cohen-Mansfield and Golander, 2011) as well as depressive symptoms, sleep disturbances, eating changes and disinhibited conducts in patients with neurocognitive disorders (Suárez-González et al., 2013; Zahodne et al., 2015).

### The Neuropsychiatric Inventory (NPI)

A subsample of patients in AD group (*n* = 14) and FTD group (*n* = 14) were assessed using the NPI scale. This scale was used as a second measure to track behavioral disturbances in disease progression of neurodegenerative disorders. The NPI (Cummins, 1997) consists of a selection of NPS and a measure of severity of each symptom. The caregiver rates each of the 12 symptoms as present or absent during the current month and, if some of those symptoms are present, they provide a measure of severity (which is tracked using a Likert scale between 1 and 3 points, with 1 being mild severity). The 12 symptoms on the NPI include behavioral/conduct symptoms (aberrant motor behavior, disinhibition, apathy), emotional-mood symptoms (euphoria, anxiety, depression, irritability), disruptive/psychotic symptoms (agitation, delusions, hallucinations) and other types of symptoms (nighttime behaviors, appetite and eating behavior disturbances). This instrument has been previously reported as efficient and reliable in tracking behavioral alterations in different neuropsychiatric disorders (Ismail et al., 2013, 2016) and neurocognitive disorders (Lai, 2014; Nowrangi et al., 2015; Ismail et al., 2016).

#### Data Analysis

Demographic and neuropsychological data for the two groups of patients (FTD and AD) were compared using one-way analysis of variance (ANOVA) and chi square tests for the categorical variables. Where indicated, Tukey’s *post hoc* tests were used to examine group differences within neuropsychological measures.

#### Analyses of Longitudinal Progression Measures and Predictive Factors

To calculate the degree of change in cognitive functioning and instrumental and physical functionality between the first and second stage of assessment, two progression indexes for cognitive performance ΔMoCA (MoCA score at stage 1 − MoCA score at stage 2) and ΔMMSE (ΔMMSE = MMSE score at stage 1 − MMSE score at stage 2) were obtained. Following the same procedure, we obtained progression indexes for functionality, ΔLawton and ΔBarthel.

In order to assess the extent to which changes in NPS predict a change in cognitive or instrumental measures in both groups, we ran independent regression models in each group using each of the above-mentioned progression indexes (i.e., ΔMMSE, ΔMoCA, ΔLawton and ΔBarthel) as dependent variables. Behavioral indexes tracked with CUSPAD (including a global score of CUSPAD obtained from sum total of each symptom of the scale) were included as independent variables in each model in order to assess whether progression in one type of symptom predicts cognitive and functional progression. To account for differences in educational level in the groups, we applied a covariance analysis to the regression models, adjusted independently for years of education (see Table 1). Effect sizes were calculated through partial eta-squared (*η*^2^). A group of similar analyses was run using the NPI scores and individual scores of each type of symptom as measures to track a broad group of behavioral disturbances in a subsample of subjects in each group (see “Instrument” section for more information on the subsample of subjects who were studied using the NPI measure). All statistical analyses were run using SPSS package version 21.0.

### Disease Progression

Previous studies have suggested that subjects who have a fall of more of four MMSE points per-year have rapidly progressive cognitive deterioration (Doody et al., 2001, 2005). Based on this approach, we have subdivided the sample of each group (AD and FTD) into subgroups according to the level of cognitive deterioration. Thus, in each clinical group, we assessed the presence of subjects with expectable deterioration (subjects with a fall of less than four MMSE points per year) and a group with subjects with fast deterioration (subjects with a fall of more than four MMSE points per year). Additionally, we assessed the presence of subjects without any level of deterioration (subjects in whom the MMSE did not change or even improved). In each of the groups, we analyzed the relationship between the presence of NPS at the early stages of the disease and cognitive and functional decline. Additionally, we performed a one-way ANOVA to analyze the magnitude of NPS between subgroups of disease progression in two groups of patients (FTD and AD).

### Mediation Analyses

We also assessed whether demographic factors (age of disease onset and disease time), medical comorbidities (presence or absence of comorbidities) and the usage of medications before diagnosis mediated the association between cognitive (ΔMoCA, ΔMMSE) and functional decline (ΔLawton, ΔBarthel) and scores of NPS tracked by CUSPAD. In addition, we assessed to what extent the cognitive decline measures mediate effects on functional decline measures and *vice versa*. To this end, we ran an independent mediation analysis for each regression model of cognitive and functional decline measures.

## Results

Results in cognitive, behavioral, and instrumental functionality measures in two stages of assessment are reported in Table 2 (see descriptive analyses in Table 2). FTD and AD groups differed in age at disease onset (*F*_(1,82)_ = 13.12, *p* < 0.001) as AD were older than FTD patients [AD = 68.6 years (SD = 8.2 years) and FTD = 60.7 years (SD = 7.5 years)]. Furthermore, FTD patients reached higher levels of formal education than AD patients [(*F*_(1,82)_ = 11.54, *p* < 0.001; AD = 11.2 years (SD = 5.5 years) and FTD = 14.8 years (SD = 6.2)]. No gender differences were found (*X*^2^_(1)_ = 0.58, *p* = 0.62).

**Table 2:** Cognitive performance.

Scales	Mean	SD
**(A) AD patients (*n* = 47)**
**Cognitive measures**
MMSE, time 1	22.9	4.8
MMSE, time 2	19.8	5.7
MoCA, time 1	17.6	5.4
MoCA, time 2	13.1	6.1
**Functionality measures**
Total Lawton, time 1	33.6	10.6
Total Lawton, time 2	24.2	9.2
Barthel Index, time 1	95.4	9.8
Barthel Index, time 2	87.8	9.9
**Neuropsychiatric symptoms**
CUSPAD, time 1
Delusions	1.4	1.1
Hallucination	0.1	0.05
Illusion	0.2	0.06
Conduct problems	1.4	1.1
Depression	2.1	0.9
Sleep problems	1.8	1.2
Eating changes	0.5	0.07
CUSPAD, time 2
Delusions	1.5	1.4
Hallucination	0.2	0.1
Illusion	0.1	0.04
Conduct problems	1.7	1.1
Depression	1.8	0.7
Sleep problems	1.1	0.6
Eating changes	0.7	0.1
**(B) FTD patients (*n* = 36)**
**Cognitive measures**
MMSE, time 1	23.1	4.9
MMSE, time 2	15.6	7.5
MoCA, time 1	17.3	5.8
MoCA, time 2	12.5	5.4
**Functionality measures**
Total Lawton, time 1	34.5	7.9
Total Lawton, time 2	27.6	7.6
Barthel Index, time 1	94.2	7.8
Barthel Index, time 2	87.2	7.1
**Neuropsychiatric symptoms**
CUSPAD, time 1
Delusions	1.9	0.8
Hallucination	0.4	0.03
Illusion	0.3	0.07
Conduct problems	2.5	1.4
Depression	1.5	0.7
Sleep problems	1.2	1.3
Eating changes	2.1	0.7
CUSPAD, time 2
Delusions	1.8	1.1
Hallucination	0.3	0.2
Illusion	0.2	0.09
Conduct problems	2.6	0.8
Depression	1.1	0.8
Sleep problems	0.7	0.1
Eating changes	1.2	1.1

*Basic and instrumental functionality and neuropsychiatric symptoms in patients with AD and FTD. Note: SD, standard deviation; AD, Alzheimer’s disease; FTD, frontotemporal dementia; CUSPAD, Columbia University Scale for Psychopathology in Alzheimer’s Disease; MMSE, Mini-Mental State Examination; MoCA, Montreal Cognitive Assessment*.

### First Stage of Assessment

Analyses did not reveal differences between FTD and AD groups in MoCA scores (*F*_(1,82)_ = 0.65, *p* = 0.81; MoCA FTD = 17.3, SD = 5.8, AD = 17.6, SD = 5.4) nor in MMSE scores (*F*_(1,82)_ = 0.88, *p* = 0.37; MMSE FTD = 23.1, SD = 4.9; AD = 22.9, SD = 4.8). With respect to functionality and instrumental measures, no differences were found between groups in the Lawton scale (*F*_(1,82)_ = 0.95, *p* = 0.33; Lawton FTD = 34.5, SD = 7.9; AD = 33.6, SD = 1.6) nor in Barthel index (*F*_(1,82)_ = 2.11, *p* = 0.13; Barthel FTD = 94.2, SD = 7.8; AD = 95.4, SD = 9.8). Regarding behavioral measures, analyses showed greater scores in the global score of CUSPAD in the FTD compared to AD (*F*_(1,82)_ = 3.95, *p* < 0.05, *η*^2^ = 0.06). In addition, the FTD group compared with the AD group showed higher scores in conduct problems (*F*_(1,82)_ = 4.22, *p* < 0.05, *η*^2^ = 0.06) and in eating change measures (*F*_(1,82)_ = 3.99, *p* < 0.05, *η*^2^ = 0.06). In contrast, the AD group had higher scores in sleep problems than in the FTD group (*F*_(1,82)_ = 3.15, *p* < 0.05, *η*^2^ = 0.06). No other comparisons reached significant values.

### Second Stage of Assessment

Analyses revealed minor MoCA and MMSE scores in FTD compared with the AD group [for MoCA (*F*_(1,82)_ = 2.93, *p* < 0.05, *η*^2^ = 0.06; MoCA FTD = 12.5, SD = 5.4; AD = 13.1, SD = 6.1)], and for MMSE (*F*_(1,82)_ = 2.76, *p* = 0.05, *η*^2^ = 0.05; MMSE FTD = 15.6, SD = 7.5; AD = 19.8, SD = 5.7). In addition, the AD group showed greater impairment in functionality than the FTD group as measured with the Lawton scale (*F*_(1,82)_ = 2.75, *p* < 0.05, *η*^2^ = 0.05; Lawton FTD = 27.6, SD = 7.6; AD = 24.2, SD = 9.2). No differences were observed in the Barthel index (*F*_(1,82)_ = 1.92, *p* = 0.16; Barthel FTD = 87.2, SD = 7.1; AD = 87.8, SD = 9.9). With respect to behavioral measures, analyses showed higher scores in depressive symptoms in the AD group compared with the FTD group (*F*_(1,82)_ = 3.76, *p* < 0.05, *η*^2^ = 0.06). In addition, results showed higher scores for eating changes in the FTD group when compared with the AD group (*F*_(1,82)_ = 3.94, *p* < 0.05, *η*^2^ = 0.06) and higher conduct problems (*F*_(1,82)_ = 2.98, *p* < 0.05, *η*^2^ = 0.06). No other comparisons reached significant values.

### Progression Indexes

A progression index for each cognitive (ΔMMSE, ΔMoCA) and functional measure (ΔLawton and ΔBarthel) was created as described above. Analyses between groups revealed greater cognitive progression in FTD compared with the AD group [ΔMoCA (*F*_(1,82)_ = 3.43, *p* < 0.05, *η*^2^ = 0.06) and ΔMMSE (*F*_(1,82)_ = 2.69, *p* < 0.05, *η*^2^ = 0.06)]. In addition, we observed a major functional progression in AD compared with the FTD group [for ΔLawton (*F*_(1,82)_ = 2.42, *p* < 0.05, *η*^2^ = 0.06)], and no differences when we analyzed progression between groups in ΔBarthel (*F*_(1,82)_ = 0.25, *p* = 0.51).

### Neuropsychiatric Symptoms and Progression in Cognitive and Functional Measures

#### AD Group

##### Analyses Using CUSPAD Scale

We ran an independent regression model using ΔMoCA as dependent measure and the CUSPAD scores at the first stage of assessment (scores for delusions, hallucinations, illusions, alterations, conduct problems, depressive and anxiety symptoms, sleep problems and eating changes) as independent measures. This model reached significant values (*F*_(1,46)_ = 4.21, *p* < 0.05, *R*^2^ = 0.19), showing that the variability in ΔMoCA was explained by the presence of depression and the score for sleep alterations. No significant effects were observed when exploring the score for other CUSPAD domains (see Table 3 for a further description of results). A similar regression model with ΔMMSE as a dependent measure of cognitive progression also reached significant values (*F*_(1,46)_ = 3.59, *p* < 0.05, *R*^2^ = 0.09). In this case, only the score for depression explained the variability of ΔMMSE (Table 3A).

**Table 3:** Independent regression model of cognitive progression (ΔMoCA and ΔMMSE) and functional physical and instrumental progression (ΔBarthel and ΔLawton, respectively) through groups of symptoms of behavior disturbance with CUSPAD at first stage in patients with AD and FTD.

Regression model	Global	Delusions	Hallucinations	Illusions	Depression	Conduct *p*.	Sleep *p*.	Eating *p*.
**(A) AD patients**
ΔMoCA (*F*_(1,46)_ = 4.21, *p* <0.05, *R*^2^ = 0.19)	*β* = 0.82 ***P* = <0.01** *η*^2^ = 0.12	*β* = 0.29 *P* = 0.19 *η*^2^ = 0.05	*β* = 0.01 *P* = 0.93 *η*^2^ = 0.01	*β* = 0.23 *P* = 0.22 *η*^2^ = 0.05	*β* = 0.48 ***P* = <0.05** *η*^2^ = 0.09	*β* = 0.16 *P* = 0.31 *η*^2^ = 0.01	*β* = 0.45 ***P* = <0.05** *η*^2^ = 0.09	*β* = 0.12 *P* = 0.34 *η*^2^ = 0.02
ΔMMSE (*F*_(1,46)_ = 3.59, *p* <0.05, *R*^2^ = 0.09)	*β* = 0.26 *P* = 0.11 *η*^2^ = 0.04	*β* = 0.25 *P* = 0.12 *η*^2^ = 0.01	*β* = 0.12 *P* = 0.49 *η*^2^ = 0.05	*β* = 0.19 *P* = 0.19 *η*^2^ = 0.05	*β* = 0.36 ***P* = <0.05** *η*^2^ = 0.02	*β* = 0.04 *P* = 0.98 *η*^2^ = 0.03	*β* = 0.22 *P* = 0.13 *η*^2^ = 0.05	*β* = 0.16 *P* = 0.31 *η*^2^ = 0.05
ΔLawton (*F*_(1,46)_ = 3.71, *p* <0.05, *R*^2^ = 0.07)	*β* = 0.11 *P* = 0.58 *η*^2^ = 0.05	*β* = 0.29 *P* = 0.09 *η*^2^ = 0.06	*β* = 0.26 *P* = 0.15 *η*^2^ = 0.05	*β* = 0.13 *P* = 0.34 *η*^2^ = 0.05	*β* = 0.33 ***P* = <0.05** *η*^2^ = 0.07	*β* = 0.10 *P* = 0.54 *η*^2^ = 0.01	*β* = 0.18 *P* = 0.38 *η*^2^ = 0.05	*β* = 0.21 *P* = 0.12 *η*^2^ = 0.05
ΔBarthel (*F*_(1,46)_ = 0.72, *p* = 0.51, *R*^2^ = 0.03)	*β* = 0.39 ***P* = <0.05** *η*^2^ = 0.09	*β* = 0.25 *P* = 0.19 *η*^2^ = 0.05	*β* = 0.23 *P* = 0.20 *η*^2^ = 0.05	*β* = 0.16 *P* = 0.21 *η*^2^ = 0.05	*β* = 0.45 ***P* = <0.05** *η*^2^ = 0.12	*β* = 0.21 *P* = 0.23 *η*^2^ = 0.05	*β* = 0.24 *P* = 0.09 *η*^2^ = 0.06	*β* = 0.03 *P* = 0.81 *η*^2^ = 0.05
**(B) FTD patients**
ΔMoCA (*F*_(1,35)_ = 7.28, *p* <0.001, *R*^2^ = 0.31)	*β* = 0.36 ***P* = <0.05** *η*^2^ = 0.12	*β* = 0.32 ***P* = <0.05** *η*^2^ = 0.11	*β* = 0.11 *P* = 0.12 *η*^2^ = 0.04	*β* = 0.03 *P* = 0.56 *η*^2^ = 0.02	*β* = 0.078 *P* = 0.31 *η*^2^ = 0.04	*β* = 0.42 ***P* = <0.001** *η*^2^ = 0.13	*β* = 0.14 *P* = 0.11 *η*^2^ = 0.04	*β* = 0.091 *P* = 0.25 *η*^2^ = 0.03
ΔMMSE (*F*_(1,35)_ = 4.41, *p* <0.05, R^2^ = 0.12)	*β* = 0.19 ***P* = <0.05** *η*^2^ = 0.06	*β* = 0.15 *P* = 0.11 *η*^2^ = 0.05	*β* = 0.08 *P* = 0.22 *η*^2^ = 0.05	*β* = 0.05 *P* = 0.61 *η*^2^ = 0.02	*β* = 0.04 *P* = 0.78 *η*^2^ = 0.04	*β* = 0.18 *P* = 0.09 *η*^2^ = 0.06	*β* = 0.11 *P* = 0.11 *η*^2^ = 0.05	*β* = 0.09 *P* = 0.26 *η*^2^ = 0.05
ΔLawton (*F*_(1,35)_ = 3.12, *p* <0.05, R^2^ = 0.06)	*β* = 0.91 ***P* = <0.001** *η*^2^ = 0.16	*β* = 0.47 ***P* = <0.05** *η*^2^ = 0.06	*β* = 0.02 *P* = 0.89 *η*^2^ = 0.05	*β* = 0.04 *P* = 0.81 *η*^2^ = 0.04	*β* = 0.21 *P* = 0.22 *η*^2^ = 0.05	*β* = 0.31 ***P* = <0.05** *η*^2^ = 0.09	*β* = 0.04 *P* = 0.81 *η*^2^ = 0.03	*β* = 0.38 ***P* = <0.05** *η*^2^ = 0.11
ΔBarthel (*F*_(1,35)_ = 0.98, *p* = 0.47, *R*^2^ = 0.03)	*β* = 0.39 *P* = 0.23 *η*^2^ = 0.06	*β* = 0.11 *P* = 0.54 *η*^2^ = 0.16	*β* = 0.17 *P* = 0.49 *η*^2^ = 0.02	*β* = 0.03 *P* = 0.88 *η*^2^ = 0.01	*β* = 0.22 *P* = 0.49 *η*^2^ = 0.02	*β* = 0.41 *P* = 0.11 *η*^2^ = 0.06	*β* = 0.25 *P* = 0.28 *η*^2^ = 0.05	*β* = 0.21 *P* = 0.33 *η*^2^ = 0.05

*Note: AD, Alzheimer’s disease; FTD, frontotemporal dementia; CUSPAD, Columbia University Scale for Psychopathology in Alzheimer’s Disease; MMSE, Mini-Mental State Examination; MoCA, Montreal Cognitive Assessment*.

A second group of regression models was run, including ΔLawton and ΔBarthel as dependent measures of functional and instrumental progression and the CUSPAD total scores, and scores in each symptom at first stage as independent measures. The model on ΔLawton reached significant values (*F*_(1,46)_ = 3.71, *p* < 0.05, *R*^2^ = 0.07), showing that the variability in ΔLawton was explained by the score for depression and the scores for sleep problems. The regression model with ΔBarthel as a dependent measure of functional physical progression and using the same independent measures did not reach significant values (*F*_(1,46)_ = 0.72, *p* = 0.51, *R*^2^ = 0.03; see Table 3A).

##### Analyses Using NPI

In a subsample of subjects (*n* = 14), we used an extra scale for tracking NPS in order to analyze the role of a broad group of behavioral symptoms in predicting cognitive and functional progression in AD patients. We ran an independent regression model using ΔMoCA as a measure of cognitive progression, and as independent measures we included the total score of NPI, and the scores in each group of symptoms of NPI at the first stage (delusions, hallucinations, euphoria, depression, anxiety, aggression, apathy, disinhibition, irritability, motor disturbance, eating changes, sleep problems). This model reached significant values (*F*_(1,13)_ = 3.14, *p* < 0.05, *R*^2^ = 0.41), showing that the variability of ΔMoCA was explained by the score of depression and the score for sleep disorders. No significant effects were observed when exploring the score for other NPI domains (see Table 4B). A similar regression model with ΔMMSE as a dependent measure of cognitive progression and the same independent measures did not reach significant values (*F*_(1,13)_ = 1.19, *p* = 0.09, *R*^2^ = 0.21). The regression models with ΔLawton as functional instrumental progression measure (*F*_(1,13)_ = 1.25, *p* = 0.55, *R*^2^ = 0.28) and ΔBarthel as functional physical progression measure (*F*_(1,13)_ = 0.97, *p* = 0.60, *R*^2^ = 0.24) did not reach significant values (see Table 4A).

**Table 4:** Significant Independent regression model of cognitive progression through groups of symptoms of behavior disturbance with NPI at first and second stage in patients with AD and FTD.

Regression model	Global	Del.	Hal.	Euph.	Dep.	Anx.	Agg.	Apa.	Dis.	Irr.	Mot. D.	Eat. C.	Sle. P.
**(A) AD patients (*n* = 14)**
ΔMoCA (*F*_(1,13)_ = 3.14, *p* < 0.05, *R*^2^ = 0.41)	*β* = 0.32 *P* = 0.10 *η*^2^ = 0.06	*β* = 0.09 *P* = 0.69 *η*^2^ = 0.04	*β* = 0.19 *P* = 0.59 *η*^2^ = 0.04	*β* = 0.08 *P* = 0.65 *η*^2^ = 0.03	*β* = 0.44 *P* <0.05 *η*^2^ = 0.07	*β* = 0.11 *P* = 0.63 *η*^2^ = 0.04	*β* = 0.04 *P* = 0.82 *η*^2^ = 0.03	*β* = 0.23 *P* = 0.22 *η*^2^ = 0.05	*β* = 0.19 *P* = 0.59 *η*^2^ = 0.04	*β* = 0.31 *P* = 0.11 *η*^2^ = 0.06	*β* = 0.08 *P* = 0.65 *η*^2^ = 0.03	*β* = 0.24 *P* = 0.21 *η*^2^ = 0.05	*β* = 0.40 ***P* = < 0.05** *η*^2^ = 0.06)
**(B) FTD patients (*n* = 14)**
ΔMoCA (*F*_(1,13)_ = 2.41, *p* < 0.05, R^2^ = 0.39)	*β* = 0.15 *P* = 0.41 *η*^2^ = 0.05	*β* = 0.47 ***P* = <0.05** *η*^2^ = 0.07	*β* = 0.21 *P* = 0.39 *η*^2^ = 0.05	*β* = 0.09 *P* = 0.72 *η*^2^ = 0.03	*β* = 0.11 *P* = 0.47 *η*^2^ = 0.04	*β* = 0.04 *P* = 0.89 *η*^2^ = 0.02	*β* = 0.17 *P* = 0.42 *η*^2^ = 0.05	*β* = 0.43 ***P* = <0.05** *η*^2^ = 0.05	*β* = 0.48 ***P* = <0.05** *η*^2^ = 0.08	*β* = 0.19 *P* = 0.31 *η*^2^ = 0.02	*β* = 0.12 *P* = 0.47 *η*^2^ = 0.02	*β* = 0.24 *P* = 0.26 *η*^2^ = 0.02	*β* = 0.22 *P* = 0.39 *η*^2^ = 0.05
ΔMMSE (*F*_(1,13)_ = 2.19, *p* < 0.05, *R*^2^ = 0.30)	*β* = 0.23 *P* = 0.39 *η*^2^ = 0.05	*β* = 0.56 ***P* = <0.05** *η*^2^ = 0.06	*β* = 0.03 *P* = 0.87 *η*^2^ = 0.03	*β* = 0.26 *P* = 0.11 *η*^2^ = 0.04	*β* = 0.29 *P* = 0.18 *η*^2^ = 0.05	*β* = 0.04 *P* = 0.83 *η*^2^ = 0.03	*β* = 0.26 *P* = 0.11 *η*^2^ = 0.04	*β* = 0.25 *P* = 0.12 *η*^2^ = 0.04	*β* = 0.48 ***P* <0.05** *η*^2^ = 0.06	*β* = 0.26 *P* = 0.11 *η*^2^ = 0.04	*β* = 0.21 *P* = 0.42 *η*^2^ = 0.04	*β* = 0.11 *P* = 0.69 *η*^2^ = 0.04	*β* = 0.38 *P* = 0.21 *η*^2^ = 0.05

*Note: AD, Alzheimer’s disease; FTD, frontotemporal dementia; NPI, neuropsychiatric inventory; MMSE, Mini-Mental State Examination; MoCA, Montreal Cognitive Assessment; Del., delusions; Hal., hallucinations; Euph., euphoria; Dep., depression; Anx., anxiety; Agg., aggression; Apa., apathy; Dis., disinhibition; Irr., irritability; Mot. D., motor disturbance; Eat. C., eating changes; Sle. P., sleep problems*.

### FTD Group

#### Analyses Using CUSPAD Scale

We ran an independent regression model using ΔMoCA as a measure of cognitive progression, and as independent measures we included the total score of CUSPAD and the scores in each group of symptoms of CUSPAD at the first stage. This model reached significant values (*F*_(1,35)_ = 7.28, *p* < 0.001, *R*^2^ = 0.31), showing that the variability, ΔMoCA was explained by the global scores for CUSPAD, the score for delusions, the score for conduct problems and the score for eating changes (see Table 3 for a further description of results). A similar regression model with ΔMMSE as dependent measure of cognitive progression and the same independent measures also reached significant values (*F*_(1,35)_ = 4.41, *p* < 0.05, *R*^2^ = 0.12; see Table 3B). In this case, only the global score of CUSPAD explained the ΔMMSE variability. The regression model with ΔLawton as dependent measure of functional and instrumental progression and CUSPAD scores as independent measures reached significant values (*F*_(1,35)_ = 3.12, *p* < 0.05, *R*^2^ = 0.06), showing that the global score of CUSPAD and the scores for conduct problems explained the ΔLawton variability. The regression model with ΔBarthel did not reach significant values (*F*_(1,35)_ = 0.98, *p* = 0.47, *R*^2^ = 0.03; see Table 3B).

#### Analyses Using NPI

In a subsample of FTD patients (*n* = 14), we assessed to what extent a broad group of behavioral symptoms predicted cognitive and functional progression using the NPI measure. The independent regression model using ΔMoCA as a measure of cognitive progression, and the total score of NPI, and the scores in each group of symptoms of NPI at the first stage as independent measures reached significant values (*F*_(1,13)_ = 2.41, *p* < 0.05, *R*^2^ = 0.39). In particular, this model showed that the variability of ΔMoCA was explained by the score of delusions, the score for disinhibition and the score for apathy. No significant effects were observed when exploring other NPI domains (see Table 4B).

A similar regression model with ΔMMSE as dependent measure of cognitive progression and the same independent measures also reached significant values (*F*_(1,14)_ = 2.19, *p* < 0.05, *R*^2^ = 0.30). In this case, the score of delusion and the disinhibition explained the ΔMMSE variability. No significant effects were observed when exploring other NPI domains. The regression models with ΔLawton as functional instrumental progression measure (*F*_(1,13)_ = 0.85, *p* = 0.65, *R*^2^ = 0.18) and ΔBarthel as functional physical progression measure (*F*_(1,13)_ = 0.93, *p* = 0.57, *R*^2^ = 0.18) did not reach significant values (see Table 4B).

### Regression Analyses According to the Subgroups of Disease Progression

#### AD Group

Among the 47 AD patients, a total of three patients did not show deterioration (6.3% of the total of the sample), a group of 38 patients showed an expectable deterioration (85.1% of the total of the sample), and four patients showed fast deterioration (8.5% of the total of the sample).

The analyses of NPS in AD subgroups only revealed differences in the depression scores at the first stage of assessment (*F*_(1,46)_ = 6.56, *p* < 0.01). No differences were observed when we analyzed the other symptoms (all *p* values above 0.3). A *post hoc* analysis (Tukey’s HSD, MS = 81.24, *df* = 46) revealed that patients who did not show deterioration presented minor depression scores compared to patients with expectable deterioration (*p* < 0.01) and patients with fast deterioration (*p* < 0.01). Additionally, patients with fast deterioration had major depression scores compared to expectable deterioration patients (*p* < 0.01).

We ran new independent regression models only in the group of patients who presented the expectable deterioration (*n* = 38) to analyze the predictive role of NPS on cognitive and functional decline in the group of patients with expectable deterioration. The regression models on this group revealed a similar pattern of results to the regression models ran with the total number of patients. In particular, the model reached significant values (*F*_(1,37)_ = 3.21, *p* < 0.05, *R*^2^ = 0.09), and showed that the variability of ΔMoCA was explained by the score for depression (*β* = 0.39, *p* < 0.05, *η*^2^ = 0.06), the score for sleep problems (*β* = 0.35, *p* < 0.05, *η*^2^ = 0.06) and the global score of CUSPAD (*β* = 0.55, *p* < 0.05, *η*^2^ = 0.06). The regression model of the ΔLawton also reached significant values (*F*_(1,37)_ = 2.99, *p* < 0.05, *R*^2^ = 0.06) and revealed that the score for depression (*β* = 0.31, *p* < 0.05, *η*^2^ = 0.06) and the scores for sleep problems (*β* = 0.29, *p* < 0.05, *η*^2^ = 0.06) explained the variability of ΔLawton. The model of ΔBarthel did not reach significant values (*F*_(1,37)_ = 0.72, *p* = 0.51, *R*^2^ = 0.03). We avoided running the regression model of ΔMMSE, considering that MMSE scores were used to categorize the subgroups of disease progression and then generate circularity.

#### FTD Group

Among the 36 FTD patients, three patients did not show deterioration (8.3% of the total of the sample), a group of 29 patients showed an expectable deterioration (80.5% of the total of the sample) and four patients showed fast deterioration (11.1% of the total of the sample). The analyses of NPS in FTD subgroups only revealed differences in the conduct problems scores at the first stage of assessment (*F*_(1,35)_ = 9.98, *p* < 0.01). No differences were observed when we analyzed other CUSPAD symptoms (all *p* values above 0.1). A *post hoc* analysis (Tukey’s HSD, MS = 81.24, *df* = 46) revealed that patients who did not show deterioration presented minor conduct problem scores compared to expectable deterioration patients (*p* < 0.001) and fast deterioration patients (*p* < 0.0001). Additionally, patients with fast deterioration had major conduct problem scores compared to expectable deterioration patients (*p* < 0.05).

Following the same procedure used in the AD group, we ran new independent regression models only in the group of patients who presented the expectable deterioration (*n* = 29). As occurred in the AD group, the regression models on this group revealed a similar pattern of results to the regression models ran with the total number of patients. In particular, the model reached significant values (*F*_(1,28)_ = 8.95, *p* < 0.001, *R*^2^ = 0.33) and showed that the global scores for CUSPAD, the score for delusions (*β* = 0.44, *p* < 0.05, *η*^2^ = 0.06), the score for conduct problems (*β* = 0.48, *p* < 0.05, *η*^2^ = 0.06) and the score for eating changes (*β* = 0.42, *p* < 0.05, *η*^2^ = 0.06) explained the variability of ΔMoCA. The regression model of the ΔLawton also reached significant values (*F*_(1,28)_ = 3.12, *p* < 0.05, *R*^2^ = 0.06) and revealed that the score for delusion (*β* = 0.45, *p* < 0.05, *η*^2^ = 0.06), the scores for conduct problems (*β* = 0.49, *p* < 0.05, *η*^2^ = 0.06) the score for eating changes (*β* = 0.39, *p* < 0.05, *η*^2^ = 0.06) and the global score of CUSPAD (*β* = 0.62, *p* < 0.01, *η*^2^ = 0.09) explained the variability of ΔLawton. The model of ΔBarthel did not reach significant values ΔBarthel (*F*_(1,28)_ = 1.28, *p* = 0.29, *R*^2^ = 0.05). We did not run the model of ΔMMSE to avoid circularity considering that this measure was used to divide the subgroups of disease progression.

### Mediation Analyses

#### AD Group

We ran independent mediation analyses to track the impact of demographical factors and medical comorbidities in the association between cognitive (ΔMoCA, ΔMMSE)-functional decline measures (ΔLawton, ΔBarthel) and NPS (tracked by CUSPAD). The regression model between ΔMoCA (dependent variable) and scores of CUSPAD (*F*_(1,46)_ = 4.11, *p* < 0.05, *R*^2^ = 0.19) showed to be mediated by medical comorbidities, age of disease, onset time of disease, ΔLawton and ΔBarthel considering that the model remained significant although the F score decreased (*F*_(1,46)_ = 2.19, *p* < 0.05, *R*^2^ = 0.05). The mediation analyses also revealed that comorbidities (*β* = 0.27, *p* < 0.05, *η*^2^ = 0.06), age of disease onset (*β* = 0.29, *p* < 0.05, *η*^2^ = 0.06) disease time (*β* = 0.49, *p* < 0.01, *η*^2^ = 0.07), ΔLawton (*β* = 0.52, *p* < 0.01, *η*^2^ = 0.07) and ΔBarthel (*β* = 0.56, *p* < 0.01, *η*^2^ = 0.07), explained the variance of the dependent measure ΔMoCA. However, no significant mediation effects were found when analyzing the usage of medications before diagnosis (*β* = 0.11, *p* = 0.26, *η*^2^ = 0.02).

A similar mediation analysis was run with the regression model of ΔMMSE (*F*_(1,46)_ = 3.59, *p* < 0.05, *R*^2^ = 0.09) and revealed a partial mediation (the regression remained significant although the F index decreased) of comorbidities, age of disease onset and disease time (*F*_(1,46)_ = 2.32, *p* < 0.05, *R*^2^ = 0.06). The mediation analyses also revealed that comorbidities (*β* = 0.26, *p* < 0.05, *η*^2^ = 0.06), the age of disease onset (*β* = 0.31, *p* < 0.05, *η*^2^ = 0.06), disease time (*β* = 0.41, *p* < 0.05, *η*^2^ = 0.06), ΔLawton (*β* = 0.58, *p* < 0.01, *η*^2^ = 0.07) and ΔBarthel (*β* = 0.32, *p* < 0.05, *η*^2^ = 0.06), explained the variability of ΔMMSE. The usage of medications before diagnosis did not reach significant mediation effect (*β* = 0.003, *p* = 0.93, *η*^2^ = 0.01).

The mediation analyses on regression model of ΔLawton (*F*_(1,46)_ = 2.24, *p* < 0.05, *R*^2^ = 0.07) also revealed a partial mediation of comorbidities, age of disease onset and disease time as revealed by the reduction of F scores in the model (*F*_(1,46)_ = 2.69, *p* < 0.05, *R*^2^ = 0.07). The mediation analyses also revealed that the presence of comorbidities (*β* = 0.29, *p* < 0.05, *η*^2^ = 0.06), the age of disease onset (*β* = 0.34, *p* < 0.05, *η*^2^ = 0.06), the disease time (*β* = 0.31, *p* < 0.05, *η*^2^ = 0.06), ΔMoCA (*β* = 0.65, *p* < 0.01, *η*^2^ = 0.07) and ΔMMSE (*β* = 0.53, *p* < 0.01, *η*^2^ = 0.07), explained the variability of ΔLawton. The usage of medications before diagnosis did not reach significant mediation effects (*β* = 0.005, *p* = 0.98, *η*^2^ = 0.01).

The model of ΔBarthel was initially non-significant (*F*_(1,46)_ = 0.72, *p* = 0.51, *R*^2^ = 0.03). The mediation analyses did not reveal mediation effects of comorbidities age of disease onset, disease time and the usage of medication before diagnosis as the model remained non-significant after the inclusion of comorbidities age of disease onset, disease time and cognitive decline measures (ΔMMSE and ΔMoCA; *F*_(1,46)_ = 1.72, *p* = 0.08, *R*^2^ = 0.03).

#### FTD Group

The mediation analyses on models of cognitive decline measures [ΔMoCA (*F*_(1,35)_ = 7.28, *p* < 0.001, *R*^2^ = 0.31) and ΔMMSE (*F*_(1,35)_ = 4.41, *p* < 0.05, *R*^2^ = 0.12)] revealed only a partial mediation of comorbidities, age of disease onset, disease time and functional decline measures (ΔLawton, and ΔBarthel), as showed by reductions in F scores [ΔMoCA (*F*_(1,35)_ = 2.67, *p* < 0.05, *R*^2^ = 0.31); and ΔMMSE (*F*_(1,35)_ = 2.44, *p* < 0.05, *R*^2^ = 0.12)]. The analyses revealed that comorbidities [ΔMoCA (*β* = 0.33, *p* < 0.05, *η*^2^ = 0.06) and ΔMMSE (*β* = 0.37, *p* < 0.05, *η*^2^ = 0.06)], disease time [ΔMoCA (*β* = 0.44, *p* < 0.05, *η*^2^ = 0.06) and ΔMMSE (*β* = 0.46, *p* < 0.05, *η*^2^ = 0.06)] and ΔLawton [ΔMoCA (*β* = 0.37, *p* < 0.05, *η*^2^ = 0.06) and ΔMMSE (*β* = 0.39, *p* < 0.05, *η*^2^ = 0.06)] explained the variability of both cognitive decline measures. The analyses on the mediation effect of age of disease onset [ΔMoCA (*β* = 0.05, *p* = 0.53, *η*^2^ = 0.05) and ΔMMSE (*β* = 0.16, *p* = 0.17, *η*^2^ = 0.05)], Barthel [ΔMoCA (*β* = 0.12, *p* = 0.22, *η*^2^ = 0.04) and ΔMMSE (*β* = 0.08, *p* = 0.34, *η*^2^ = 0.05)] and the usage of medication [ΔMoCA (*β* = 0.06, *p* = 0.92, *η*^2^ = 0.01) and ΔMMSE (*β* = 0.12, *p* = 0.64, *η*^2^ = 0.01)] did not reach significant effects.

The mediation analyses on the model of ΔLawton (*F*_(1,35)_ = 2.89, *p* < 0.05, *R*^2^ = 0.06) revealed only partial mediation effects due to comorbidities, age of disease onset and disease time as the regression models showed reductions in F score (ΔLawton (*F*_(1,35)_ = 2.22, *p* < 0.05, *R*^2^ = 0.06). The mediation analyses showed that the disease time (*β* = 0.44, *p* < 0.05, *η*^2^ = 0.06), ΔMoCA (*β* = 0.34, *p* < 0.05, *η*^2^ = 0.06) and ΔMMSE (*β* = 0.31, *p* < 0.05, *η*^2^ = 0.06) explained the variability of ΔLawton. Additionally, analyses revealed that no comorbidities (*β* = 0.13, *p* = 0.34, *η*^2^ = 0.04), no age of disease onset (*β* = 0.11, *p* = 0.39, *η*^2^ = 0.04) and no usage of medication before diagnosis (*β* = 0.07, *p* = 0.78, *η*^2^ = 0.01) reached significant beta values to explain the variability of ΔLawton.

The mediation analyses on ΔBarthel did not reveal mediation effects as the initial model between CUSPAD scores and ΔBarthel showed to be non-significant (*F*_(1,35)_ = 0.98, *p* = 0.47, *R*^2^ = 0.03) and remained non-significant after the inclusion of comorbidities, age of disease onset, disease time, cognitive decline measures and the usage of medications before diagnosis (*F*_(1,35)_ = 0.44, *p* = 0.71, *R*^2^ = 0.01).

## Discussion

In this study, we have evaluated the role of the presence of NPS in predicting cognitive and functional decline in AD and FTD. Our results indicate that during the initial stages of disease, NPS affect in particular and differentiated ways, the cognitive and functional disease progression for each type of neurocognitive disorder. In the case of AD, the results show that the presence of depressive symptoms as well as sleep problems had a higher predictive value for cognitive alterations than other NPS. In addition, the scores for depressive symptoms predict functional and instrumental progression. Conversely, the results from the FTD group showed that the global scores of behavioral alterations tracked by CUSPAD, the scores of delusions, conduct problems and eating changes at early stages of assessment had a higher predictive value for cognitive and functional progression. Together, our results highlighted the importance of tracking since early stages of disease assessment the presence of different types of NPS, as those symptoms directly impact disease course.

In our study, the sample of subjects in each group coursed with an expectable pattern of disease progression (patients who had a fall of around four MMSE points per-year). In the AD and FTD groups, more than 80% of cases presented an expectable pattern of deterioration.

Crucially, our results showed that subgroups of patients according to the degree of disease progression also differed in the magnitude of NPS symptoms at early stages of assessment. In particular, in the AD group, the patients with fast and expectable deterioration presented at early stages of assessment major depressive scores compared to patients without deterioration. In the same line, the FTD patients with fast deterioration and expectable deterioration showed major scores of conduct problems compared to patients with deterioration at early stages of assessment. However, our results are far from being conclusive as we have few patients in the subgroup of fast deterioration and the group without deterioration. Future studies should explore the differences in NPS in subgroups of disease progression by including more patients in each subgroup. The Independent regression analyses only in the subgroup of patients with expectable deterioration revealed a similar pattern of results as those observed in the total of sample in each group. This pattern confirms that the results were reliable, and results were not due to out of range effects in subjects with atypical disease course.

Additionally, the impact of NPS at first stage of assessment on cognitive and functional decline was corroborated by a group of extra analyses performed in a subsample of AD and FTD patients. By using the NPI (Cummins, 1997), a sensible instrument to track a broad spectrum of behavioral alterations (affective changes, psychotic symptoms, anxiety symptoms, behavioral control alterations, apathy, sleep disorders, eating changes and motor disturbances among others), we confirmed that the early behavioral alterations predict cognitive and functional decline. In agreement with the results found using the CUSPAD scores, we observed that depression and sleep disorders tracked by NPI predict cognitive decline in AD. This pattern of results affirms the preeminence of affective and somatic symptoms in predicting a poor disease course in AD. Besides, in FTD, the presence of delusions, apathy and disinhibition tracked by NPI predict cognitive decline. Crucially, with the usage of NPI, we were able to track two nuclear symptoms for FTD diagnosis (Zamboni et al., 2008; Rascovsky et al., 2011). Our results showed that apart from being crucial for diagnosis, those symptoms also have a role in determining the disease course.

In agreement with previous studies, our results revealed mediation effects of demographical factors (in particular age of disease onset and disease time; Borroni et al., 2008; Choi et al., 2016) and comorbidities (Kansal et al., 2016; Eldholm et al., 2018) on the association between NPS and cognitive and functional decline in both groups. However, the described pattern of results in regression models remained significant after mediation analyses, suggesting that the relationship between early NPI and cognitive and functional decline are not completely explained by comorbidities and demographical factors. In addition, our results showed a mutual mediation effect of cognitive and functional decline measures supporting previous studies revealing that both cognitive and functional measures are interdependent (Cornelis et al., 2017).

Concerning the potential factors that could mediate the association between NPI and cognitive and functional decline measures, our study presents some limitations. First, although we found no mediation effects on cognitive and functional disease progression related to the usage of medications before diagnosis, we did not include further analysis on the impact of pharmacological and non-pharmacological interventions to alleviate NPS during the first and second stages of assessment on the cognitive-functional progression. Previous studies have shown that pharmacological interventions, rehabilitation plans and person-centered interventions could impact disease progression in both AD and FTD (Atri, 2011; Fazio et al., 2018). Future studies should assess to what extent different pharmacological interventions could reduce the disease progression in both AD and FTD. Second, we did not further assess the mediation effect of different types of comorbidities on cognitive and functional decline in neurodegenerative disorders. Previous studies have shown a strong impact of vascular complications on cognitive and functional decline (Sena et al., 2015; Janota et al., 2016). Future studies should control for both AD and FTD the impact of comorbidities in generating and maintaining the NPI and to what extent a combination of those factors accelerate the disease progression.

Previous studies have demonstrated the importance of depression in determining the progression towards a major neurocognitive disorder (Brendel et al., 2015; Zahodne et al., 2015; Kaup et al., 2016). A meta-analysis of 12 studies published in 2013 (Gao et al., 2013) showed that depression was a major risk factor for incidence of dementia (including AD, vascular dementia and any dementia) and Mild cognitive impairment (MCI). Our study adds new information on the role of different types of NPS as predictors of the course of AD. The presence of sleep problems predicted a worsening cognitive function in AD patients. Our results are in line with these studies, and also indicate that sleep alterations at the first stages of disease could also predict functionality impairments (Palmer et al., 2018). Furthermore, when depressive symptoms and sleep problems are present during the first stages of AD, they are predictive of major impairments in instrumental functionality 2.5 years into disease progression, which differs from other studies (Hallikainen et al., 2018) in which delusions, agitation, and aberrant motor behavior at the time of diagnosis predicted later AD progression.

In addition, we demonstrate that the presence of depressive symptoms in AD is associated with worse cognitive prognosis (Teng et al., 2007). This is important, since it suggests that specific monitoring of depression should have a greater value in predicting deterioration in AD. Our findings differ from a previous study (Peters et al., 2015), where NPS were predictors of greater progression to severe neurocognitive disorder and death, and affective symptoms were associated with death only (Rabins et al., 2013). In addition, it also differs from other studies where psychotic symptoms, agitated behavior, and aberrant motor behavior were associated with rapid disease progression (Buccione et al., 2007; Hallikainen et al., 2018). The presence of at least mild or one clinically significant NPS at baseline (compared with no symptoms) predicted both severe dementia and death (Rabins et al., 2013; Rosenberg et al., 2013), but this was not discriminated by NPS, as it was done in our study.

Other studies have commented on having at least mild or one clinically significant NPS at baseline (compared with no symptoms) and predicted both severe dementia and death (Peters et al., 2015; Rabins et al., 2013; Rosenberg et al., 2013), but this was not discriminated by NPS as was done in our study.

Using FDG PET, it is possible to determine that individuals with basic mild cognitive impairment with depressive symptoms have a higher load of B-amyloid with hypermetabolism in brain areas compared to non-depressive individuals (Ng et al., 2017). This could be related to the presence of active inflammation, resulting in a high-risk group for greater progression to a major neurocognitive disorder (Brendel et al., 2015).

This could be related to the presence of active inflammation, resulting in a high-risk group for greater progression to a major neurocognitive disorder (Brendel et al., 2015). One study reported that patients with depression in AD had more medial temporal lobe atrophy (MTA) than patients with AD without depression (Dhikav et al., 2014), and a post-mortem study revealed that AD patients with a history of depression had more neuritic plaques and neurofibrillary tangles in the hippocampus than those without a history of depression (Rapp et al., 2008). Therefore, in patients with neurocognitive disorders, depression could cause greater accumulation of *β*-amyloid, atrophy and inflammation leading to further deterioration, as demonstrated by our findings. Therefore, in patients with neurocognitive disorders, depression could cause greater accumulation of *β*-amyloid and inflammation, leading to further deterioration, as demonstrated by our findings. However, given that patients with depression experience alteration in volition and motivation, it is not clear whether progression is biased by depressive symptoms; in other words, it is not clear to what extent alterations and cognitive and functional progression are explained by the fact that these patients have depressive symptoms. Future studies should explore this further. Although there is no consensus on the interaction between cognition and affective symptoms in AD (Wilson et al., 2002; Rapp et al., 2011), previous studies suggest that depression does affect progression at least in patients with mild cognitive impairment (Kaup et al., 2016). Our results agree with earlier findings that revealed the role of depression in predicting cognitive declining. Thus, emotional and affective reactions associated with the neuropathological changes may manifest as an affective syndrome, signaling an impending decline in functional abilities (Palmer et al., 2018). Our findings implied that depression treatment might be applied to prevent or delay the occurrence and development of AD in patients with MCI.

However, given that patients with depression experience alteration in volition and motivation, it is not clear whether progression is biased by depressive symptoms; in other words, it is not clear to what extent alterations and cognitive and functional progression are explained by the fact that these patients have depressive symptoms. Future studies should explore this further. Although there is no consensus on the interaction between cognition and affective symptoms in AD, previous studies suggest that depression does affect progression at least in patients with MCI (Kaup et al., 2016). Our results are in agreement with earlier findings that revealed the role of depression in predicting cognitive declining. However, in our study, the presence of depressive symptoms also impacts the functionality. Although it is possible that functional impairments are directly related to cognitive decline, the role of depressive symptoms in explaining impairments in functionality could also be associated with the fact that depressive symptoms also affect volitional and motor skills, which are necessary for functional autonomy. Altogether, our results show that it is of vital importance to assess depressive symptoms more carefully in patients with AD. Our findings support the necessity for new studies that evaluate whether an intervention on depression can modify the cognitive course of the underlying condition.

Previous studies have reported a high prevalence of sleep alterations in different neurodegenerative disorders (Mander et al., 2016). In fact, a possible association between behavioral pathways that include sleep alterations and neuropathological changes was recently demonstrated (Ehrenberg et al., 2018). Sleep-wake disturbances have been associated with the formation of amyloid plaques, and with the accumulation of the amyloid-*β* (A*β*) peptide (Lim et al., 2014). Our results are in line with these studies, and also indicate that sleep alterations at the first stages of disease could also predict functionality impairments (Palmer et al., 2018).

The presence of higher global scores in the scale tracking different types of NPS (CUSPAD), along with conduct problems and delusions predicts a worsened progression of the cognitive state and functionality in the FTD group. The role of NPS in FTD has shown to be nuclear and constituted of an accurate diagnosis for a behavioral variant of FTD, but also in linguistic variants (Gorno-Tempini et al., 2011; Rascovsky et al., 2011). Conduct problems, in particular those related to apathy and disinhibition, are present from the very early stages of disease and many times in prodromes (Bott et al., 2014). In addition, it has recently been reported that the type of the first symptom in FTD, in particular the predominant presence of apathy or disinhibition, is related to a particular pattern of neurocognitive impairment (Santamaría-García et al., 2016). In this study, we add new data on the role of NPS in FTD by showing that the progression of a mixed group of NPS (general alteration of NPS and particularly, the presence of conduct problems) leads to a worse cognitive and functional prognosis.

Contrary to what we observed in AD, depression is not a strong predictor for cognitive and functional progression in FTD. Poor control of behavior in general could be explained by a pattern of classic neurocognitive alterations in FTD, characterized by alterations in key structures for behavioral regulation, including frontal, temporal, and insular structures (Ibañez and Manes, 2012; Baez et al., 2017). These findings could also suggest that NPS are another face of the neurocognitive degeneration seen in FTD. In this sense, a progression of cognitive, functional, and behavioral alterations in FTD is generated by classic neurocognitive changes in this condition. Another explanation to be explored in future studies is whether the presence of NPS generates a worsening of the cognitive and functional profile in FTD. This scenario would agree with disease progression studies in FTD that show an increase and mixed pattern of NPS, and an association between NPS and prognosis in this condition. Therefore, a broader exploration of the role of NPS in cognitive function in FTD, particularly whether NPS worsen the course of the disease or they are simply a consequence of the general frontotemporal neurocognitive alteration observed in FTD, is needed.

Furthermore, we observed that the presence of delusions is predictive of cognitive impairment in FTD. Although the presence of psychotic symptoms is more prevalent in neurocognitive disorders associated with Lewy Bodies (Cagnin et al., 2013; Gossink et al., 2017; Pezzoli et al., 2017), previous studies have also reported that FTD patients could be affected by delusions (Gossink et al., 2017). Delusions are especially pertinent as they are less widely recognized as harbingers of structural brain disease and more likely to lead to psychiatric misdiagnosis. Delusion mechanisms are likely to involve neural networks in the frontal and temporal lobes that are particularly vulnerable in FTD (Omar et al., 2011). Our results also reveal that a major presence of delusions could be a bio-behavioral predictor of cognitive decline in FTD (Mendez et al., 2008).

Finally, results in the FTD group also demonstrate that the presence of eating changes at the first stage of disease predicts cognitive and functional impairments in these patients. Eating abnormalities including appetite changes, increased carbohydrate craving, and changes in food preference are present in up to 60% of patients with frontotemporal dementia (FTD), and are one of the core symptoms required for the diagnosis of behavioral variant FTD (bvFTD; Rascovsky et al., 2011; Ahmed et al., 2015, 2018). Arguably, eating behavior alterations in FTD could be explained by altered frontal and temporal lobe volume, as well as impairments in networks linking those structures to the striatum and orbitofrontal cortices (Ahmed et al., 2018). Crucially, the impairments in these fronto-temporal and insular networks are at the core of behavioral and cognitive alterations in FTD. Our results indicate that the presence of eating changes at the first stages of disease could be a sensitive bio-behavior marker of cognitive and functional impairment in FTD.

Conduct problems and delusions are related to changes in frontotemporal and insular networks (Ibañez and Manes, 2012), and one might argue that eating changes could be considered as initial clinical footprints of the classical neurocognitive impairment pathway reported in FTD.

Our findings support the need for further studies exploring the differential role of various types of symptoms in predicting cognitive and functional changes in neurocognitive disorders. In particular, it is important to elucidate the role of apathy, disinhibition and irritability in predicting the general course of the disease progression, with the presence of particular pathways of neural atrophy and cognitive impairments of neurocognitive disorders. Similarly, it is essential to refine techniques for the observation and measurement of NPS during the diagnostic process, as well as in the follow-up of neurocognitive disorders. To this effect, it is necessary to include information provided by family members, companions, and caregivers, as well as the assessment of NPS clinicians, in order to improve symptom evaluation. Taken together, our results highlight the need to better and more closely monitor NPS in neurocognitive disorders, along with studies that can determine if the presence of NPS can have a generative (causal) role in cognitive alterations. If so, new studies should also consider whether an effective treatment of behavioral disturbances would change the clinical course of these conditions.

Our results might be elucidating several aspects of the same problem. It is well known that changes in behavior are associated with changes in cognition and functionality. In some cases, cognitive alterations are found in patients without behavioral alterations and may be a predictive factor. Despite this, our study shows that behavioral progression is a determining factor that should be monitored and evaluated when analyzing the progression of neurocognitive disorders.

Therefore, NPS are much more than specifiers in neurocognitive disorders and are likely to play a central role in neurodegenerative diseases. The results of our study also support further studies to establish whether there are neurocognitive biomarkers associated with the progression of NPS in neurocognitive disorders and to evaluate the impact that adequate treatment and control of NPS can have on the functional and cognitive progression of these pathologies.

Analyses in the subsample of AD patients assessed with NPI revealed that depressive but not anxiety symptoms significantly predict cognitive decline. This pattern of results is in agreement with results reported with the CUSPAD. Contrary to the CUSPAD, the NPI is more selective in tracking particular types of symptoms. Although in our study the assessment with NPI revealed significant predictor effects for depressive symptoms, it is unclear to what extent the negative results for other behavioral symptoms are due to a restricted number of subjects assessed with this instrument. Future studies should assess the impact of behavioral symptoms on cognitive and functional decline by using different types of measures able to track a broader profile of behavioral, affective and cognitive symptoms.

In addition, it would be useful to assess the predictive role on cognitive and functional decline in neurodegeneration of other cognitive and psychological factors, including the copying styles, problem solving skills and personality traits, which have beeb shown to be relevant factors in predicting disease course in neurodegenerative disorders (Irish et al., 2012a,b; D’Iorio et al., 2018).

In this study, we have tried to fully analyze to what extent a broad group of behavioral symptoms could impact different cognitive and functional decline measures. A potential limitation of our study is that we have tested different regression models with a considerable number of predictors and outcomes. Evidently, the presence of a major number of factors and levels of analyses could increase error rates and false positive effects. However, we considered that there are some attenuators for this issue. First, we were interested in assessing the impact of NPS on disease progression following an approach that did not follow any *a priori* assumptions on NPS associated to disease progression. In this sense, the inclusion of variables of levels of analyses were needed to maintain the independence of assumptions in this topic. Second, we have tried to control the effects of multidimensionality and false positives by using effect sizes and *post hoc* analyses of each contrast when it was appropriate. Future studies should assess the multidimensionality of behavioral alterations in disease progression of AD and FTD by using another statistical approach including multidimensional reduction following principal component analyses or machine learning methods.

## Conclusion

In this study, we demonstrate that the progression of behavioral disturbances is a determining factor in predicting functional and cognitive deterioration in patients. In particular, we observed that the presence of general behavioral alterations, delusions and eating changes predicts functional and cognitive impairment in FTD. In contrast, in patients with AD, the progression of depression as well as sleep changes are two crucial behavioral alterations that predict changes in the course of the condition. Taken together, our results highlight the importance of detecting and monitoring NPS in patients with different types of neurocognitive disorders.

## Ethics Statement

Participants in this study provided verbal informed consent to be enrolled in this study. All patient caregivers provided written informed consent according with the Helsinki declaration. This study was approved by The Ethics Committee of the Pontificia Universidad Javeriana and the Hospital Universitario San Ignacio in Bogotá (Colombia). Additionally, we ran a secondary analysis of the information obtained in clinical records of patients. In particular, we assessed the scores of the results of NPI from that clinical records. All information obtained of the clinical records were treated ensuring confidentiality and privacy of identification information.

## Author Contributions

JSE and HS-G participated in design and conceptualization of the study, data acquisition, analysis and interpretation of the data, writing and revision of the manuscript. JB and ÁP participated in the data acquisition, data analysis and revision of the manuscript. DM, PR and VP-S participated in the design and conceptualization of the study and provided revisions of the manuscript. All authors contributed equally in the preparation of the manuscript. The manuscript was approved by all coauthors.

## Conflict of Interest Statement

The authors declare that the research was conducted in the absence of any commercial or financial relationships that could be construed as a potential conflict of interest.
